# Stable epidermal electronic device with strain isolation induced by in situ Joule heating

**DOI:** 10.1038/s41378-021-00282-x

**Published:** 2021-07-24

**Authors:** Zihao Wang, Qifeng Lu, Yizhang Xia, Simin Feng, Yixiang Shi, Shuqi Wang, Xianqing Yang, Yangyong Zhao, Fuqin Sun, Tie Li, Ting Zhang

**Affiliations:** 1 i-lab, Key Laboratory of Multifunctional Nanomaterials and Smart Systems, Suzhou Institute of Nano-Tech and Nano-Bionics (SINANO), Chinese Academy of Sciences (CAS), 398 Ruoshui Road, Suzhou, Jiangsu 215123 P. R. China; 2 Nano Science and Technology Institute, University of Science and Technology of China, 96 Jinzhai Road, Hefei, Anhui 230026 P. R. China; 3 School of Computer Science & School of Cyberspace Science, XiangTan University, Yuhu District, Xiangtan, Hunan 411105 P. R. China

**Keywords:** Electrical and electronic engineering, Nanosensors

## Abstract

Epidermal electronics play increasingly important roles in human-machine interfaces. However, their efficient fabrication while maintaining device stability and reliability remains an unresolved challenge. Here, a facile in situ Joule heating method is proposed for fabricating stable epidermal electronics on a polyvinyl alcohol (PVA) substrate. Benefitting from the precise control of heating locations, the crystallization and enhanced rigidity of PVA are restricted to desired areas, leading to strain isolation of the active regions. As a result, the electronic device can be conformably attached to skin while showing negligible degradation in device performance during deformation. Based on this method, a flexible surface electromyography (sEMG) sensor with outstanding stability and highly comfortable wearability is demonstrated, showing high accuracy (91.83%) for human hand gesture recognition. These results imply that the fabrication method proposed in this research is a facile and reliable approach for the fabrication of epidermal electronics.

## Introduction

Flexible electronics are considered the next revolution in the electronics industry due to their potential applications in areas unreachable with rigid devices^1–6^. As a vital part of flexible electronics, epidermal electronics can be essentially applied in the area of health monitoring and human-machine interfaces (HMIs)^7,8^. To achieve highly comfortable wearability and outstanding device performance, a stretchable and ultraflexible epidermal electronic device is, by tuning the material modulus and device structures, is desirable to achieve conformable contact with human skin^4,9^. Generally, there are two strategies to fabricate epidermal electronics. The first method is to fill polymer composites with conductive materials, such as carbon nanotubes, graphene, and metal nanowires^10–16^, while the other method uses metals with designed structures, including serpentine and accordion bellow shapes^17–19^. Metal-based devices take advantage of their electrical conductivity compared with their polymer-based counterparts. However, the brittleness and modulus mismatch between metals and soft substrates is a challenge that cannot be ignored in device design and fabrication^20,21^.

Strain isolation, a strategy to protect the active region by modifying the intrinsic rigidity contrast between metal-based devices and soft substrates, offers a possible solution to overcome the above problems^22–25^. Usually, delicate mechanical design is required to achieve reliable strain isolation, which includes assembling inorganic components on surface relief patterns rather than on flat surfaces^26,27^, embedding hard platforms within soft substrates^22^, and inserting a soft layer between devices and substrates^25^. However, there the use of strain isolation strategies in device manufacture is considerably complex. For example, strain isolation typically demands the use of transfer printing, in which active components in a designed layout are deposited on a silicon wafer and then transferred onto an elastomeric substrate in two separate steps^28^. Due to complexities in the preparation process, it is still a challenge to fabricate a thin (submicrometer) metal-based device to satisfy the condition of conformal contact^7,29^.

Herein, inspired by the mechanism of strain isolation, we propose a facile in situ Joule heating method to directly fabricate stable epidermal electronics on PVA substrates. The intrinsic stiffness contrast in the polymer substrate obtained by Joule heating offers a simple yet robust mechanism without resorting to transfer printing. With an in situ Joule heating treatment, programmable changes in the crystallinity distribution can be achieved in the coplanar PVA substrate, resulting in a designed swelling rate and stiffness of the treated membrane. When the polymer substrate contact skin, the region without treatment absorbs moisture from the skin, and the membrane swells, resulting in a thinner substrate and perfect adapting to skin folds. To demonstrate the advantage of this method, metal-based surface electromyography (sEMG) sensors with low impedance, highly comfortable wearability, and good conformal adhesion properties were fabricated. In addition, human hand gesture classification based on the fabricated sEMG sensors was implemented, and a high classification accuracy of over 91.83% was achieved.

## Results and discussion

### Properties of PVA membranes at different thermal treatment temperatures

**Fig. 1:**
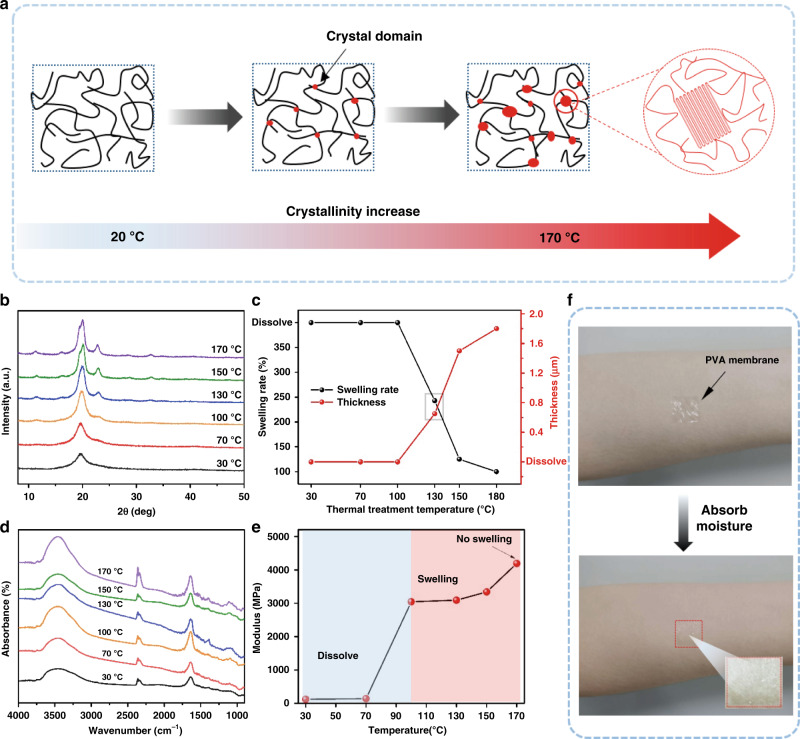
Properties of PVA at different thermal treatment temperatures. **a** Crystallinity of PVA increases with increasing heat treatment temperature. **b** XRD spectra of PVA at different heat treatment temperatures. The gradual decrease in the FWHM for the diffraction peak at 22.7° suggests an increase in the crystallinity of PVA. **c** IR spectra of PVA at different heat treatment temperatures. The increase in the band at 1141 cm^−1^, which is attributed to symmetric C–C, in the FTIR spectra is another characteristic of PVA membrane treatment at different temperatures. **d** Changes in the thickness and swelling rate of PVA membranes treated at different temperatures. The swelling rate decreases with increasing treatment temperature, which is consistent with the XRD results. **e** The modulus of PVA increases with increasing treatment temperature. **f** PVA membranes (thermal treatment at 130 °C) absorb moisture from the environment (such as skin and air), and the membrane can conformably contact skin

In this research, a nontoxic, easily processable, and biocompatible PVA membrane, was employed as the substrate. Previous reports indicated that the hydrogen bonds formed between PVA molecules could hinder crystallization of the membrane^30^; thus, a decrease in the swelling rate of PVA is associated with an increase in PVA crystallinity^31^. The crystallization of PVA can be enhanced by removing water through thermal treatment. Fig. 1a shows a schematic diagram of the crystallization process during the thermal treatment of PVA. At high temperature, the gradual loss of water in the PVA membrane leads to a decrease in the number of hydrogen bonds, and PVA crystallization occurs during the cooling process. By precise control of the thermal treatment temperature, different degrees of PVA crystallinity can be realized, as indicated by the XRD patterns in Fig. 1b. The gradual decrease in the full width at half maximum (FWHM) of the diffraction peak at 19.8° with increasing annealing temperature is an indicator of the improvement in crystallinity. In addition, the intensity of the absorption peak at 1141 cm^−1^, which is attributed to the asymmetric stretching vibration of the C–C bond^31,32^, significantly increases with increasing annealing temperature, as illustrated in Fig. 1c. These results confirm that the degree of crystallinity of the PVA membrane can be precisely controlled by tuning the thermal treatment temperature, thus further influencing the swelling rate of the PVA membrane when exposed to moisture, as shown in Fig. 1d. When the treatment temperature is lower than 100 °C, the PVA substrate completely dissolves in water. As the temperature increases, the swelling rate of the substrate decreases, and almost no swelling occurs when the annealing temperature reaches 170 °C. As a result, the thickness of the substrate after swelling decreases with decreasing annealing temperature, as indicated by the red curve in Fig. 1d. In addition, as illustrated in Fig. 1e and Fig. S1, the modulus of the PVA membrane increases rapidly when the temperature reaches 100 °C and becomes saturated thereafter. All these results indicate that it is possible to control the thickness and Young’s modulus of PVA membranes by modulating the thermal treatment temperature. Figure 1f shows an example showing that after annealing at 130 °C for 30 min, the PVA membrane is able to conformably adapt to skin folds after absorbing moisture from the epidermis and environment.

### Fabrication procedure of the metal layer on a PVA membrane

**Fig. 2:**
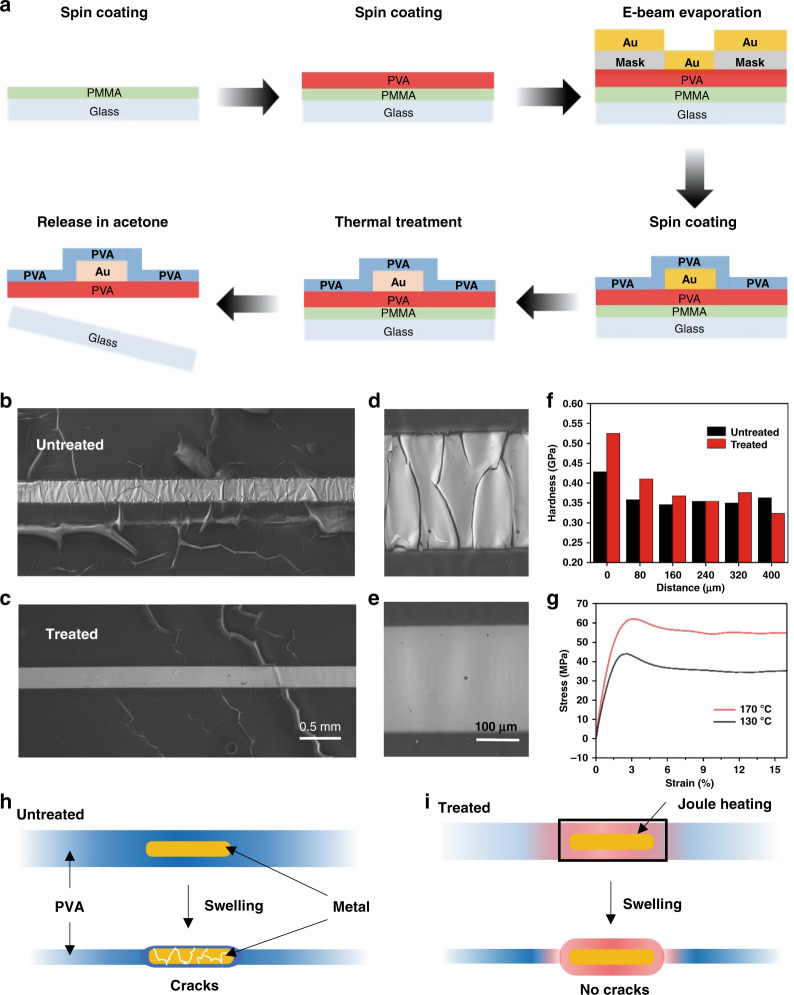
Schematic diagram showing the fabrication procedure of a metal layer on a PVA membrane. **a** Fabrication process of patterning metal layers on a PVA membrane. **b** The PVA membrane swells when exposed to a moisture-rich environment, and cracks appear for the metal line without any heat treatment. **c** Negligible deformation of the metal line occurs when it is subjected to heat treatment. SEM images of **d** untreated and **e** heat-treated metal lines. The influence of heat treatment on the hardness of the PVA substrate is shown in **f**, where a distance of 0 is the edge of the metal line. **g** Modulus of the PVA membrane with thermal treatment temperatures of 130 °C and 170 °C. It is verified that the modulus of PVA surrounding the Joule heating metal layer is enhanced but changes little in other areas. Schematic diagrams of the cross-section of a PVA membrane with a metal layer. After absorbing moisture, **h** cracks appear on the metal layer without thermal treatment, but **i** the metal layer with thermal treatment remains complete and protected by the lack of swelling of the PVA membrane

Based on this, we fabricated a patterned metal layer on a PVA substrate and optimized the stress and modulus mismatch between the metal and PVA membrane through precise Joule heating treatment. As demonstrated in Fig. 2a, layers of polymethyl methacrylate (PMMA) and PVA were initially spin-coated on a glass substrate and cured at 130 °C for 30 min. Then, chromium (10 nm) and gold (100 nm) were deposited on the substrate as a metal layer, which was patterned by electron beam evaporation through a hard mask, followed by spin-coating another layer of PVA on top as a protection layer. Afterward, a Joule heating process was carried out only on the PVA/metal layer/PVA area with a custom system, consisting of heating probes, a microscope, and a DC power supply, as shown in Fig. S2. After thermal treatment, the PVA membrane-sandwiched metal layer exhibits a high modulus and loses its swelling property, while other areas without annealing can still absorb moisture and swell when exposed to a moisture-rich environment.

Figure 2b shows the as-prepared patterned metal layer (metal line, 300 µm) on the same PVA membrane without thermal treatment. Clear cracks appear on the metal, and the metal line is destroyed (Fig. 2d). In contrast, the metal layer on the PVA membrane after annealing remains intact, and the regions near the metal show good swelling properties and can adhere conformably to arbitrary surfaces (Fig. 2c and e). After thermal treatment, the stiffness of the PVA substrate increased, as reflected by its measured hardness increase (Fig. 2f). These results are also consistent with the modulus change of PVA with thermal treatment at 130 °C and 170 °C (Fig. 2g). In addition, thermal treatment of metal areas can decrease their strain. This can be explained by Hooke’s law:

$${\Delta}l = \frac{{F_Nl}}{{EA}}$$

where Δ*l* is the elongation of the solid material, *l* is the original length of the material, *F*_*N*_ is the force applied to the material, *E* is the modulus of the material and *A* is the cross-sectional area of the material. As illustrated in Fig. 2h, without thermal treatment, the thickness of the PVA substrate decreases. Therefore, the metal layer in the active region cannot be effectively protected by the PVA substrate, and obvious cracks appear. However, when the substrate is annealed with the in situ Joule heating treatment, the PVA substrate in the active region does not significantly swell when exposed to a moisture-rich environment, and no cracks appear on the metal layer (Fig. 2i). The decreased thickness of the untreated region and the increased modulus in the treated region both contribute to the decreased strain in the thermally treated area. Therefore, the thermal treatment area can be protected by strain isolation, and self-adhesion of the substrate is achieved after swelling.

### Structural design and mechanical performance of the sEMG sensors

**Fig. 3:**
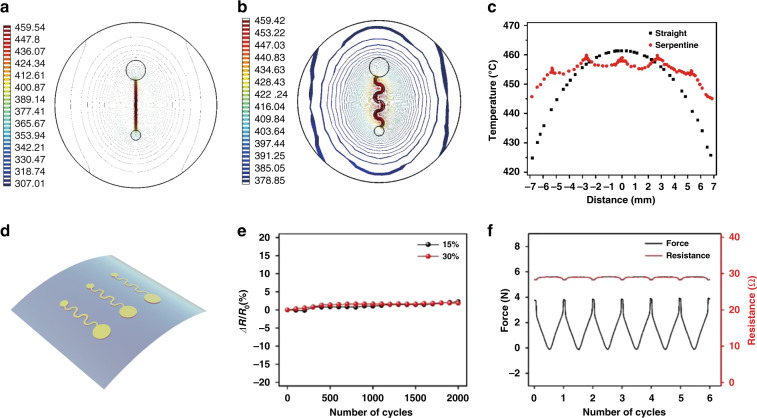
Structural design and mechanical performance of the sEMG sensors. **a**, **b** Heat distribution isotherm diagrams for sEMG sensors with a straight line and a serpentine metal line, respectively. A more uniform temperature distribution can be achieved for the serpentine metal line based on simulations. **c** The difference in the temperature can reach 36 °C for the sensor with a straight design, which is ~20 °C higher than that of the serpentine metal sensor. **d** Schematic illustration of an sEMG sensor with a PVA substrate. **e** Resistance change of the sEMG sensors under 15 and 30% stretching on a skin replica for 2000 cycles. **f** A resistance change within 2 Ω is measured during each stretching cycle (30%)

Based on the above results, sEMG sensors with long-term reliability can be fabricated with the proposed method. In addition, to improve device performance, we applied the finite element method (FEM) to simulate the temperature field distribution and optimize the structural design of the sensors. The temperature distribution of a straight-line sensor is shown in Fig. 3a, where the highest temperature appears in the center and decreases rapidly when moving away from the center. A significant temperature difference of ~35.6 °C between the center and margin areas is measured for the straight-line structure (black curve in Fig. 3c), which leads to a large difference in mechanical strength among these areas. In addition, a large temperature difference affects the swelling rate of the substrate and influences its adhesion to skin, thus degrading and damaging the metal layer due to the bulging of the PVA substrate (Fig. S3). In comparison, the temperature difference of the metal layer between the center and the ends for the serpentine structure design is only 14.7 °C, which is much lower than that of the straight structure (Fig. 3b and c). This observation was further verified by experiments, where a temperature variation of 15.8 °C was obtained, as shown in Fig. S4.

Based on the simulation results, we fabricated a serpentine-shaped sEMG sensor on a PVA substrate (Fig. 3d). A photograph and the structure design of the prepared sensor are shown in Figs. S5 and S6, respectively. To investigate the stretchability of the sensor, we fixed the device on a Mark-10 digital force gauge and measured its resistance fluctuation during stretching. When stretching the sensor to 15 or 30%, the resistance of the device for both cases only changes within 3% after 2000 cycles (Fig. 3e). In addition, during a single stretching cycle with a stretching rate up to 30% and a stretching force reaching 3.8 N, the resistance change of this device is 2 Ω (Fig. 3f). These results indicate that this sensor can endure the impact of people’s daily actions; thus, stable sEMG signals can be obtained using this sensor.

### Electrical performance of the sEMG sensors

**Fig. 4:**
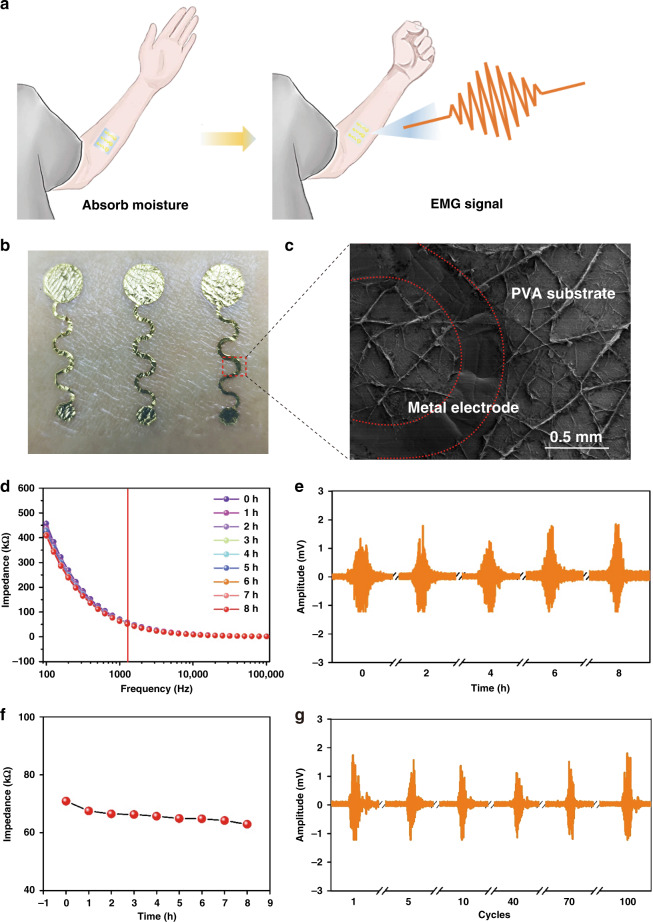
Electrical performance of the sEMG sensors. **a** After absorbing moisture from the environment, the sensors can conformably fit skin folds and record sEMG signals. **b** Image of the sEMG sensors attached to human skin. **c** SEM image of a sensor that has been peeled off of skin. The area circled in red is the metal layer, which combines well with the substrate. **d** No significant increase in the impedance of the sensors is observed when attached to skin for 8 h. **e** sEMG signals measured with the sensors. **f** No significant changes in the impedance of the sensors at 1 kHz are observed. **g** sEMG signals for a series of muscle contractions followed by rest measured with the as-prepared sensors attached to the wrist

Next, we placed the sEMG sensor on skin in a moisture-rich environment and collected EMG signals (Fig. 4a). As expected, the PVA substrate absorbed moisture and became thinner allowing the sensor to conformally attach to the skin (Fig. 4b) due to swelling of the membrane. As illustrated in Fig. 4c, the sensor adapts well to skin folds, and no obvious cracks are observed.

Benefitting from their excellent mechanical stability, the sEMG sensors exhibit good electrical performance. First, impedance, which is an important factor that influences sEMG signals, was measured. Compared with commercial gel sEMG sensors, the fabricated sEMG sensors exhibit better long-term stability^33^, which is of great importance in long-term monitoring^29^, despite a slight increase in impedance. As shown in Fig. 4d–g, negligible changes in impedance are observed after 8 h of use. The decrease in impedance from 75 to 65 kΩ at a 1 kHz frequency in the first hour can be attributed to the initial adaptation of the sensor to skin folds. During the 8 h, the sensors were bent 100 times, and no significant degradation of the EMG signals is observed. These results further verify the stability of the device.

### Gesture classification of multichannel sEMG signals

To verify the advantages of the sEMG sensors, we compared the recognition rate of captured signals between the prepared sEMG sensors and commercial sEMG sensors. Eight sensors were wrapped around a wrist at a sampling rate of 1000 Hz, among which six were test sensors and the other two were a reference sensor and ground sensor. A volunteer was asked to repeatedly perform six defined gestures in sequence 100 times, and a total of 600 corresponding gesture signals were recorded.

**Fig. 5:**
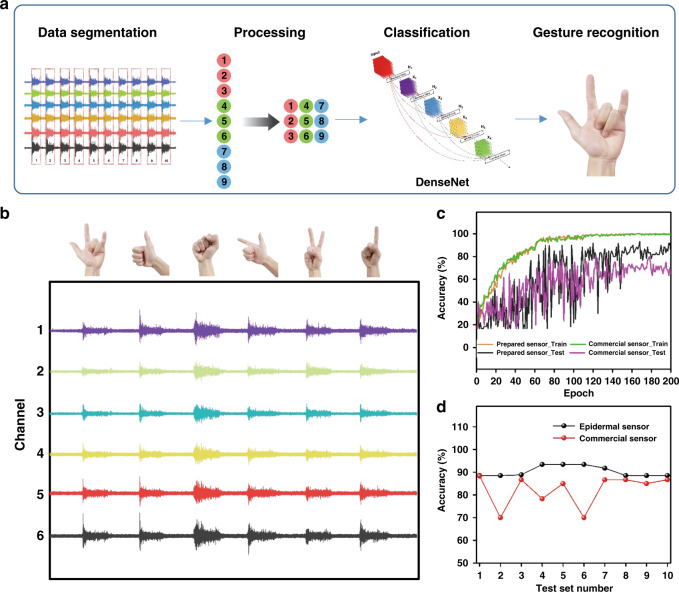
Gesture classification of multichannel sEMG signals. **a** Structure of the program. The sEMG signal is segmented into ten parts, and one gesture is included in each part. Then, the data are processed into a 64*64 matrix. Next, the matrix is input into DenseNet to classify the gestures. Finally, the gesture is recognized. **b** sEMG signals for six different gestures. **c** Training process of the data: the accuracy of the training increased as the number of epochs increased. The accuracy of the epidermal sensor was higher than that of the commercial gel sensor. **d** Recognition accuracy with different test sets. The accuracy of the prepared sensor was better than that of the commercial sensor while also being more stable

Then, PyTorch software, including a manual segmentation module, a data preprocessing module, and a classifier module, was used to analyze and recognize gesture signals, as shown in Fig. 5a. The segmentation module starts by manually labeling the peaks of the sEMG wave and subsequently obtains 4500 values as segmented gesture data. Then, the above 4500 values are compressed into a 64*64*6 matrix through the data processing module. After that, the classifier module inputs the matrix into DenseNet100^34^. After 200 epochs of training, a classifier model and parameters based on sEMG gesture signals are obtained. Figure 5b presents the sEMG signals of different hand gestures extracted through the six channels. The signal-to-noise ratio (SNR) of the sEMG signals during the test time is illustrated in Fig. S7. With increasing operation time, the SNR of the sEMG signals acquired by the commercial sensors decreases from 24.3 to 6.5. In contrast, the SNRs of the sEMG signals acquired by the prepared sensor are within the range of 19.8–23.4. As shown in Fig. 5c, the recognition rate slowly increases as training progresses for both the commercial and fabricated sensors in this experiment, and the recognition rate becomes saturated at a certain value. This result indicates that the model used in this research was convergent and that overfitting was avoided.

The accuracy for each individual test is presented in Fig. 5d. According to the statistical results, the average accuracy rates for the commercial and fabricated sensors were 82.33% and 91.83%, respectively. The above results also demonstrate that in the case of long-term use, the fabricated sensors degrade more slowly than commercial sensors, resulting in a higher recognition rate.

## Conclusion

In this work, strain isolation theory was applied to epidermal electronics, and a new method based on this theory was proposed to fabricate sEMG sensors on PVA membranes. With the metal devices undergoing a precise Joule heating process, the stiffness of the active area was enhanced, while the adhesion of the substrate was not affected. The optimal structure of the sEMG sensors was designed using the FEM. The sensors showed outstanding performance in terms of wearable comfort and stability compared with commercial gel sensors. Furthermore, to demonstrate the potential application of the sensors in HMIs, the sensors were shown to be able to recognize the sEMG signals of human gestures using machine learning algorithms with a high accuracy of 91.83%. These results indicate that this method provides an idea to fabricate epidermal electronic devices in an efficient but robust way.

## Experimental section

### Preparation of the PVA membrane

A 12% PVA (PVA-124, Aladdin) solution was obtained by dissolving PVA-124 in deionized water and stirring for 30 min at 100 °C. PMMA (P141443, Aladdin) solution was obtained by dissolving PMMA powder in ethyl lactate (Aladdin) at a 30% weight ratio and stirring for 50 min at 150 °C. First, the PMMA solution (2 ml) was coated on a 2-inch glass substrate by spin coating (3000 rpm, 30 s) and baked at 70 °C on a hot plate for 10 min, which was used as a sacrificial layer. Then, a PVA layer (2 ml) was coated on the PMMA layer by spin coating (4000 rpm, 30 s) and baked at 130 °C on a hot plate for 30 min.

### Material properties

XRD was performed to investigate the crystallinity of PVA before and after heat treatment (Bruker D8). FTIR spectra were collected via a Thermo Nicolet iN 10 spectrometers with a laser operating at 77 K in a liquid nitrogen environment. Tensile tests were carried out using a universal material testing machine. A hardness test was carried out by a nanoindentation tester in continuous stiffness measurement mode. The distance between each test point was 80 µm, and the depth limit of every test point was 300 nm.

### Structural design of the sEMG sensors

The appropriate structure of the sEMG sensors was investigated by performing FEM and the AC/DC module and heat transfer module were required to simulate electrical heat generation and heat transfer. Joule heating treatment models were established by drawing a geometric model of the sensor in simulation software and defining boundary conditions to simulate the real situation. The temperature distribution in the model can be more uniform by optimizing the size (line width, arc radius, etc.) of the model.

### Fabrication of the sEMG sensors

A metal mask was laminated on the PVA membrane, and Ti and Au layers with thicknesses of 10 nm and 100 nm were deposited using E-beam evaporation (Ei-5z, ULVAC). The deposition rate was set at 1.5 Å per second. Then, another PVA layer was coated on the metal as a protective layer (spin-coating, 3000 rpm, 30 s), followed by annealing at 130 °C for 20 min. After that, a custom heating system was used to carry out the Joule heating process with an applied voltage of 8 V for 10 min. Finally, the PVA membrane with sEMG sensors was peeled off from the glass by dissolving the PMMA in acetone (immersed in acetone at 60 °C for 20 min).

### Characterization of the devices

The temperature distribution test was carried out by a thermocouple thermometer (Kaipusen TES 1310) with the temperature probe fixed on the probe table. The temperature distribution of the metal line was monitored by moving the temperature probe to different places and testing the temperature.

The mechanical performance was investigated using a Mark-10 digital force gauge. The details are as follows: the sEMG sensor was placed on a skin replica made of PDMS (Dow Corning Sylgard 184), the sensor was stretched to the programmed length (15 and 30%), and the impedance change of the sensor was investigated using a KEITHLEY 2602 instrument.

Skin/electrode interface impedance measurements were taken using an electrochemical workstation (Gamry Reference 600+) with frequencies ranging from 100 to 10^5^ Hz. The spacing between each electrode was maintained at 50 mm. EMG signals were recorded by multiplex bioelectric data acquisition equipment (ZJE-II) at a sampling rate of 1000 Hz. Commercial sensors with diameters of ~50.5 mm were purchased from Liveyai Medical, which is a traditional Ag/AgCl gel electrode for disposable use.

### Gesture recognition

Eight sensors were wrapped around a wrist in a ring, six of which provided valid signals; the sampling frequency was 1000 Hz. After one day of collection, the wearer repeated one of the six gestures, each movement lasted for approximately five seconds, and the same movement was repeated ten times before changing to the next gesture. Ultimately, 100 samples were collected for each gesture, reaching a total of 600 gesture samples. PyTorch was used to complete all experiments on a desktop computer with an NVIDIA 1060. As shown in Fig. 5a, to recognize the sEMG gesture signal, the program included a manual segmentation module, a data preprocessing module, and a classifier modul34e. The detailed steps of the segmentation module consist of first manually annotating the peaks of the sEMG wave and extracting the subsequent 4500 values as the segmented gesture data. The detailed steps of the data preprocessing module consist of compressing the above 4500 values into a 64*64 matrix and then obtaining a 64*64*6 matrix by stacking the six channels of data. The classifier module inputs the 64*64*6 matrix into DenseNet100. After two hundred epochs of training, a classifier model and parameters based on the sEMG gesture signals are obtained. Considering the limited amount of experimental data, a tenfold cross-validation method was used to calculate the signal recognition rate 10 consecutive times.

## Supplementary information

Supporting Information
