# Supplementary material for: Stable epidermal electronic device with strain isolation induced by in situ Joule heating
Source: Microsyst Nanoeng. 2021 Jul 24;7:56. doi: 10.1038/s41378-021-00282-x (PMC8433187; doi:10.1038/s41378-021-00282-x)
Supplement: Supplementary file 1 — Supporting Information [file 41378_2021_282_MOESM1_ESM.pdf]

Supporting Information

Stable Epidermal Electronic Device with Strain Isolation Induced by In-situ Joule Heating

Zihao Wang[1,2], Qifeng Lu[1], Yizhang Xia[3], Simin Feng[1], Yixiang Shi[1], Shuqi Wang[1], Xianqing Yang[1], Yangyong Zhao[1], Fuqin Sun[1], Tie Li[1], Ting Zhang[1]*

[1] i-lab, Key Laboratory of Multifunctional Nanomaterials and Smart Systems, Suzhou Institute of Nano-Tech and Nano-Bionics (SINANO), Chinese Academy of Sciences (CAS), 398 Ruoshui Road, Suzhou, Jiangsu 215123, P. R. China.
[2] Nano Science and Technology Institute, University of Science and Technology of China, 96 Jinzhai Road, Hefei, Anhui 230026, P. R. China.
[3] School of Computer Science & School of Cyberspace Science, XiangTan University, Yuhu District, Xiangtan, Hunan 411105, P. R. China

*Corresponding author: tzhang2009@sinano.ac.cn

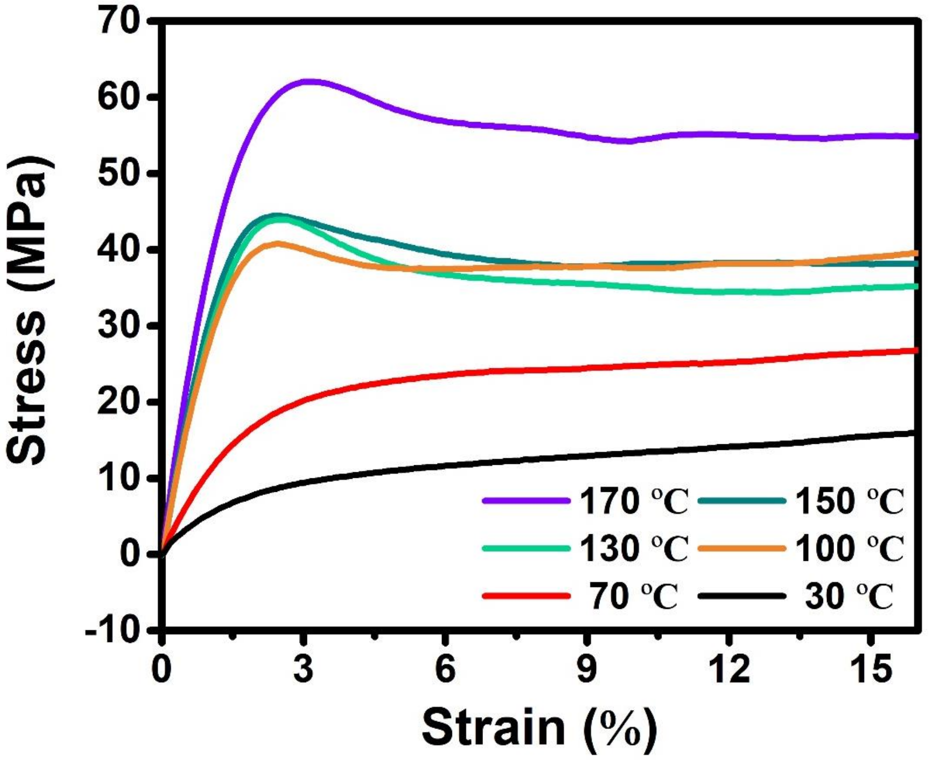

Fig. S1 Stress-strain curve of PVA with heat treatment from 30 ℃ to 170 ℃. The modulus of the PVA membranes increases with temperature increases.

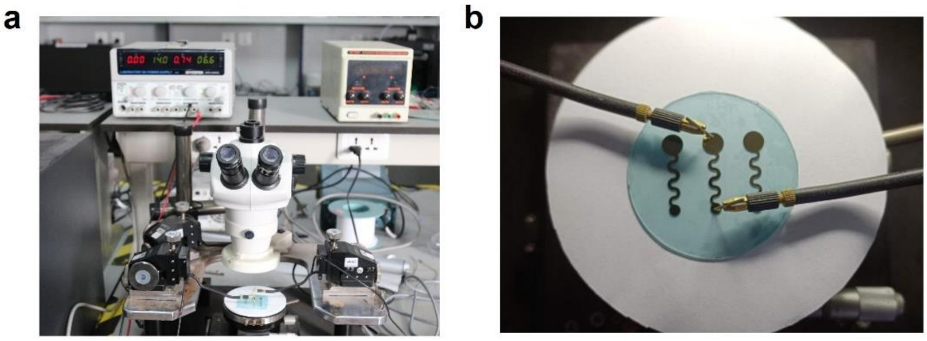

Fig. S2 Home-made Joule heating system. a) The system is mainly composed of three parts: a DC power supply, a probe station with a microscope and heating probes. b) The sensors were heated by the heating probes.

The homemade Joule heating system is mainly composed of three parts: heating probes, a probe station with a microscope and a DC power supply. The heating probes are powered by the DC power supply, which can be used to adjust the current or voltage to satisfy the needs of devices. The microscope can help in contacting the heating probes to the functional areas and heating the functional areas precisely.

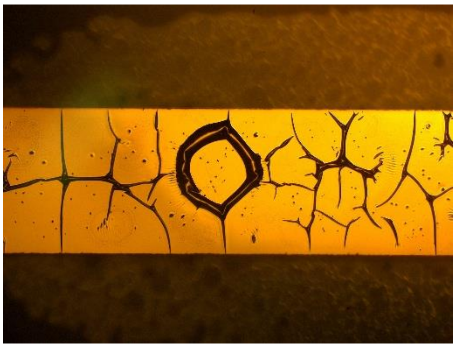

Fig. S3 Without any structure design, the PVA substrate will bulge in the central area, and the metal line will break when the local temperature is too high.

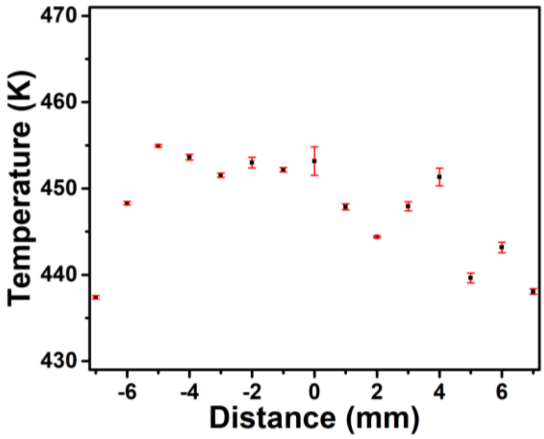

Fig. S4 Temperature distribution of the serpentine structure sensor. The temperature difference on it is similar to the theoretical difference.

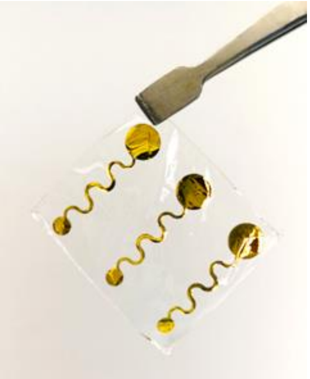

Fig. S5 Photograph of prepared sEMG sensors after peeling off from glass.

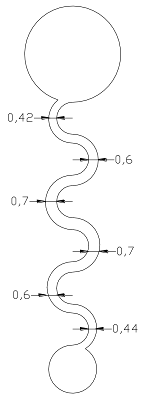

Fig. S6 Structure design of sEMG sensors. From the centre to the ends of the serpentine structure, the width of the metal line decreases gradually.

The serpentine structure of the sensors contributed to the stretchability of the sensors. Moreover, as illustrated in Fig. S6, the widths and curvatures of the metal line in different areas are not equal. According to the formula for the resistance,

$$R = \rho \frac{L}{S}$$

where R is the resistance of the conductor, ρ is the resistivity of the conductor, L is the length of the conductor, and S is the cross-sectional area of the conductor. The resistance of the conductor is affected by its conductivity, length and cross-sectional area. Therefore, the resistances in different sections of the metal line are not equal. The resistance in the middle part of the metal line is lower than that at the end points. According to Joule's Law,

$$Q = I^2 Rt$$

where Q is the heat generated by the conductor in calories, I is the current of the conductor, R is the resistance of the conductor, and t is the power-on time. Therefore, a higher resistance will generate more heat under the same current, so the middle part will generate less heat than the end points. The boundary conditions in the centre area and end areas of the sensors are different, and heat dissipation in the central area is harder than that at the ends. Reducing heat generation in the central area will be helpful

for balancing the temperature distribution, so the temperature distribution on the sensors will be more even.

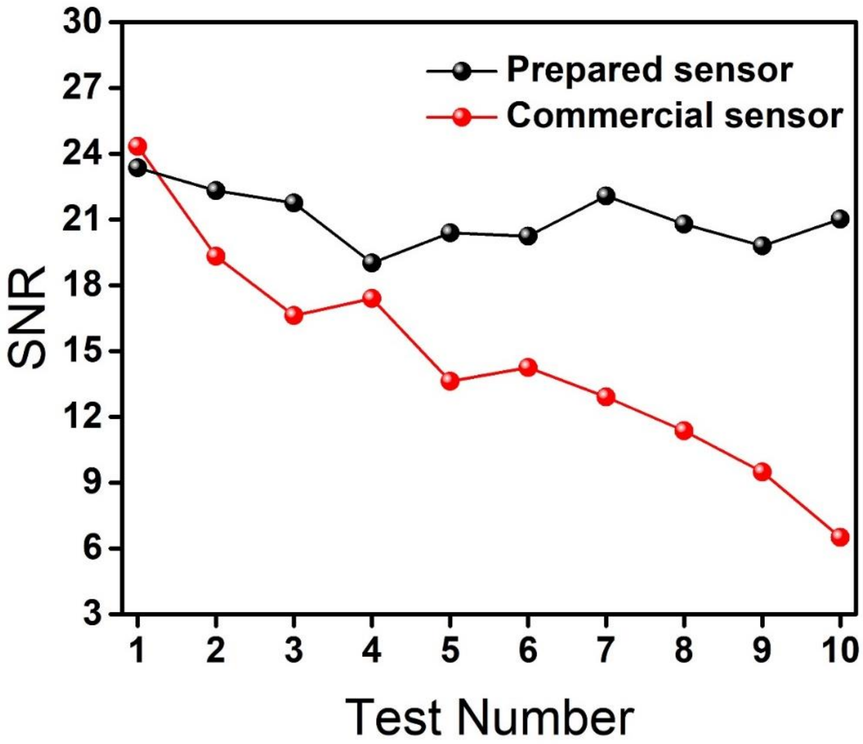

Fig. S7 SNR of the sEMG signals using for gesture recognition, sEMG signals acquired by the prepared sensor shown negligible change in SNR, while the commercial one shown an instability in SNR.
